# Synergistic effects of antibiotics and fucoidan on dual-species *Staphylococcus aureus* and *Acinetobacter baumannii* biofilm in diabetic rat wound models

**DOI:** 10.1371/journal.pone.0342905

**Published:** 2026-02-19

**Authors:** Mohsen Nazari, Mohammad Taheri, Fatemeh Nouri, Maryam Bahmanzadeh, Mohammad Yousef Alikhani

**Affiliations:** 1 Department of Microbiology, School of Medicine, Hamadan University of Medical Sciences, Hamadan, Iran; 2 Department of Pharmaceutical Biotechnology, School of Pharmacy, Hamadan University of Medical Sciences, Hamadan, Iran; 3 Department of Anatomical Sciences, School of Medicine, Hamadan University of Medical Sciences, Hamadan, Iran; 4 Fertility and Infertility Research Center, Avicenna Institute of Clinical Sciences, Hamadan University of Medical Sciences, Hamadan, Iran; 5 Infectious Disease Research Center, Avicenna Institute of Clinical Sciences, Hamadan University of Medical Sciences, Hamadan, Iran; University of New South Wales, AUSTRALIA

## Abstract

**Background:**

Chronic diabetic wounds are often complicated by biofilm-forming, antibiotic-resistant pathogens such as *Staphylococcus aureus* and *Acinetobacter baumannii*, which delay healing. This study evaluated the synergistic effects of gentamicin and imipenem in combination with fucoidan, a sulfated polysaccharide from brown seaweed, against dual-species biofilms in a diabetic rat wound model.

**Methods:**

Methicillin-resistant *S. aureus* (MRSA) strain 6 and *A. baumannii* strain 1, isolated from diabetic foot ulcers, were used to establish dual-species biofilms in vitro and in vivo. Excisional wounds were created in male Wistar rats with streptozocin-induced type II diabetes and infected with the biofilms. Rats received daily treatments of gentamicin, imipenem, their combination, or the triple combination with fucoidan. Outcomes assessed included bacterial load (CFU/g), biofilm formation, expression of biofilm-related genes (*icaA* and *bap* by real-time PCR), wound size, and histological healing parameters.

**Results:**

The triple therapy demonstrated the strongest antibacterial effect, reducing bacterial load by more than 4 log₁₀ CFU/g compared to controls (p < 0.005). Real-time PCR revealed significant downregulation of *icaA* in *S. aureus* (threefold decrease) and *bap* in *A. baumannii* (fourfold decrease) relative to antibiotic-only groups (p < 0.005). Histology showed accelerated wound contraction and complete re-epithelialization by day 14 with the triple combination, whereas monotherapy or dual antibiotics led to delayed healing and persistent inflammation.

**Conclusions:**

Fucoidan enhances the efficacy of gentamicin and imipenem against biofilm-associated infections and promotes diabetic wound healing. This combinatorial approach offers a promising strategy for managing chronic, biofilm-infected wounds and combating antibiotic resistance.

## 1. Introduction

Chronic wounds, particularly in diabetic patients, are often complicated by persistent infections that are difficult to treat due to the formation of bacterial biofilms [1]. *Staphylococcus aureus* and *Acinetobacter baumannii* are among the most common pathogens responsible for these infections, and their ability to form biofilms on wound surfaces further complicates treatment strategies [2,3]. Biofilm formation creates a protective environment for bacteria, making them significantly more resistant to antibiotics and immune system clearance compared to their planktonic counterparts [4,5]. In *S. aureus*, biofilm formation is primarily mediated by the *icaA* gene, which encodes an enzyme involved in the synthesis of polysaccharide intercellular adhesin (PIA). This polysaccharide is crucial for bacterial adhesion and the structural integrity of biofilms [6]. Similarly, in *A. baumannii*, the *bap* (biofilm-associated protein) gene encodes a large surface protein that facilitates the initial adhesion of bacteria to surfaces and contributes to cell aggregation, promoting biofilm formation [7]. The production of PIA in *S. aureus* and the *bap* protein in *A. baumannii* significantly enhances the pathogenicity and persistence of these bacteria in chronic infections, making them difficult to treat [8].

The increasing prevalence of antibiotic-resistant pathogens has exacerbated the challenge of treating biofilm-related infections [9]. Resistance to multiple antibiotic classes, particularly in biofilm-forming strains, severely limits the effectiveness of conventional antibiotic therapies. Both *S. aureus* and *A. baumannii*, major contributors to chronic wound infections, have acquired resistance to a wide range of antibiotics, complicating their management [10]. This growing resistance underscores the urgent need for novel therapeutic approaches to treat infections, especially those associated with biofilms in chronic wounds.

Fucoidan, a sulfated polysaccharide derived from brown seaweed, has shown promising antimicrobial properties [11]. Studies have demonstrated its potential to disrupt biofilms and inhibit bacterial adhesion [12]. Fucoidan’s ability to break down biofilm matrices and enhance antibiotic penetration has sparked interest in its use as an adjunctive treatment to restore or augment the effectiveness of conventional antibiotics [13]. However, given that fucoidan is a high-molecular-weight polysaccharide, its direct penetration into mature biofilms remains a subject of debate. Current evidence suggests that fucoidan may not necessarily penetrate biofilms deeply but instead acts by destabilizing the extracellular polymeric substance (EPS) through electrostatic interactions and matrix disruption, thereby indirectly facilitating antibiotic diffusion [14]. These effects are supported primarily by in vitro and ex vivo studies, and direct visualization or quantitative confirmation of fucoidan penetration within biofilm structures is still limited. This approach could be particularly beneficial in addressing the challenges posed by antibiotic-resistant, biofilm-forming pathogens [15]. In this study, we investigate the synergistic effects of imipenem and gentamicin in combination with fucoidan against dual-species biofilms formed by *S. aureus* and *A. baumannii* in a diabetic rat wound model.

Building upon our previous in vitro research, where we explored the effects of imipenem, gentamicin, and fucoidan on biofilm formation by *S. aureus* and *A. baumannii*, this study extends the investigation to an in vivo diabetic rat model [16]. Here, we examine the impact of the antibiotic-fucoidan combination on biofilm disruption, bacterial load, and wound healing. Additionally, we will study the molecular mechanisms underlying biofilm formation by analyzing the expression of the *icaA* gene in *S. aureus* and the *bap* gene in *A. baumannii*. These genes play critical roles in biofilm development, and understanding their regulation in response to combination therapy will help inform more effective strategies for combating chronic, biofilm-related infections. Ultimately, this work aims to provide valuable insights into novel therapeutic strategies for treating biofilm-associated infections in diabetic wounds, with the potential to improve clinical outcomes, enhance antibiotic efficacy, and combat the growing challenge of antibiotic resistance.

## 2. Materials and methods

### 2.1. Bacterial strains

In this study, we utilized methicillin-resistant *S. aureus* strain 6 and *A. baumannii* strain 1, both of which were previously investigated in vitro [16]. These bacterial strains were isolated from diabetic foot ulcers. The Ethics Review Board of Hamadan University of Medical Sciences, Hamadan, Iran approved the present study (Ethical approval code: IR.UMSHA.REC.1401.809). All

### 2.2. Materials

Polyvinyl chloride (PVC) coverslips, clear, non-curling, 0.20 mm thick, and 22 mm wide, were cut into 12 mm² pieces and sterilized under UV light for biofilm induction on wound surfaces. Fucoidan (a sulfated polysaccharide), gentamicin and imipenem, were purchased from Sigma-Aldrich (USA). To prepare the fucoidan stock solution, 100 mg of fucoidan powder was dissolved in 1000 mL of distilled water (0.1 mg/mL).

### 2.3. Animals

Male Wistar rats (8–10 weeks old, weighing 170–200 g) were used in this study. Animals were housed under a 12-hour light/dark cycle with free access to standard laboratory chow and water ad libitum. All experimental procedures were conducted in accordance with the ARRIVE guidelines and were approved by the Ethics Committee of Hamadan University of Medical Sciences (Ethical approval code: IR.UMSHA.REC.1401.866) [17].

To prevent the transmission of bacterial pathogens, infected and non-infected animals were maintained in separate rooms. All personnel involved in animal handling followed strict aseptic precautions to minimize the risk of cross-contamination.

Humane endpoints were established in accordance with institutional animal care regulations. Rats were monitored at least twice daily for signs of pain, distress, or illness, including weight loss exceeding 20%, immobility, hunched posture, labored breathing, or inability to eat or drink. Animals meeting any of these criteria were immediately euthanized by an overdose of ketamine (200 mg/kg) and xylazine (20 mg/kg) administered intraperitoneally. All surgical and wound procedures were performed under anesthesia using ketamine (80 mg/kg) and xylazine (10 mg/kg) to minimize discomfort. No unanticipated deaths occurred during the experiment.

### 2.4. In-vitro dual-species biofilm formation assay

The biofilm-forming ability of *S. aureus* strain 6 and *A. baumannii* strain 1 was assessed using an in vitro biofilm formation assay. First, overnight bacterial cultures were adjusted to a cell density of 1 × 10^6 CFU/mL in Luria-Bertani (LB; Merck, Germany) broth. A 100 µL aliquot of a 1:1 pre-mixed suspension of the two strains (total 100 µL per well, with equal proportions of each species) was then added to the wells of a microtiter plate and incubated for 24 hours at 37°C. Biofilm formation was evaluated using the crystal violet binding method [18].

After incubation, planktonic cells were carefully removed using a micropipette, and the wells were washed three times with sterile distilled water. Next, 20 µL of 0.1% (w/v) crystal violet, filtered through a 0.44 µm filter, was added to each well and allowed to stain for 10 minutes. The wells were subsequently rinsed with 10 mM potassium phosphate buffer and air-dried for 15 minutes. To solubilize the dye, 100 µL of 96% (v/v) ethanol was added to each well. After 15 minutes, the contents were mixed thoroughly, and the absorbance was measured at 570 nm using a Micro ELISA Auto Reader (BioTek, Germany).

### 2.5. Dual-species formation on coverslips

To initiate the experiment, *S. aureus* strain 6 and *A. baumannii* strain 1 were each introduced into sterilized Luria-Bertani (LB) broth within separate culture bottles and incubated at 37°C. Following overnight incubation, the bacterial cultures were adjusted to an optical density of 0.01 at 600 nm (OD₆₀₀) [19]. Subsequently, 5 mL of each adjusted culture was transferred into fresh LB medium and placed in a shaker incubator set to 37°C at approximately 210 rpm for a period of three hours. This allowed the bacteria to reach the mid-logarithmic growth phase.

At this stage, the cultures were distributed into sterile test tubes. UV-sterilized polyvinyl coverslips were inserted into the tubes, fully immersed in the medium and oriented vertically at a 90-degree angle to the base of the tubes. The samples were then incubated again at 37°C for 24 hours to promote bacterial adhesion and biofilm formation [20]. Following this incubation, the coverslips were gently removed and rinsed with 10 mM potassium phosphate buffer to remove non-adherent, planktonic cells.

### 2.6. Induction of type II diabetes in rats

Type II diabetes was induced in rats using a streptozocin-based method [21]. In summary, the animals were deprived of food for 12 hours prior to treatment. Each rat then received an intraperitoneal injection of nicotinamide at a dose of 240 mg/kg. Fifteen minutes later, streptozotocin was administered intraperitoneally at a dose of 100 mg/kg (Sigma, St. Louis, MO). Seventy-two hours after the streptozotocin injection, the animals underwent another 12-hour fasting period, after which fasting blood glucose levels were measured. Rats exhibiting glucose levels above 150 mg/dL were considered diabetic and included in the experimental group. No deaths were recorded in the streptozotocin-treated group.

### 2.7. Excision wound and dual-species biofilm formation

A skin area on the dorsal region of the rat was selected for the wound and depilated using a hair-removal cream. After depilation, the area was cleaned thoroughly with normal saline, and the animals were observed for 12 hours for any signs of irritation or inflammation caused by the hair removal cream. The following day, the rats were anesthetized with a ketamine (80 mg) and xylazine (10 mg) cocktail, administered intraperitoneally [22].

Once anesthetized, a full-thickness excision was made to the depilated skin area, and any bleeding was carefully controlled using sterile absorbent cotton. A 100 µL broth culture containing 10^6^ CFU/mL of *S. aureus* strain 6 and *A. baumannii* strain 1 was applied to the wound site. A coverslip with the dual-species biofilm was then gently placed over the wound and secured with surgical adhesive tape. No further treatment was administered during the next 48 hours. After this period, the coverslip and tape were carefully removed using surgical forceps, and photographs were taken to document the infection and biofilm formation on the wound. A thin biofilm layer had formed over the excised area [22].

Some rats were euthanized with an overdose of ketamine and xylazine, and the biofilm layer was carefully removed. A portion of the biofilm was used for bacterial load determination, while the remaining sample was fixed in 10% neutral formalin for further analysis.

The experiment included five groups of diabetic rats (eight animals per group) to evaluate the effects of various treatments on wound healing in the presence of a dual-species biofilm. Group 1 served as the control group and received no treatment following wound infection. Group 2 was treated with gentamicin at a concentration of 128 µg/mL, Group 3 received imipenem at 128 µg/mL, Group 4 received a combination of gentamicin (32 µg/mL) and imipenem (64 µg/mL), and Group 5 was treated with gentamicin (32 µg/mL), imipenem (32 µg/mL), and fucoidan (62.5 µg/mL). The concentrations of antibiotics and fucoidan were chosen based on the minimum biofilm inhibitory concentrations (MBICs) determined in our previous in vitro study, and on prior in vitro synergy analyses [11]. All treatments were applied topically once daily for 12 days, starting on day 2 post-wounding. Each solution was freshly prepared and uniformly distributed over the wound surface using a sterile applicator to ensure complete coverage.

Wound area measurements were conducted on days 1, 4, 7, and 14 post-wounding initiation. A sterile millimeter-scale ruler was placed adjacent to each wound as a calibration reference. The wound area (mm²) was calculated by analyzing the digital images using ImageJ software (National Institutes of Health, USA), with the calibration scale used to convert pixel dimensions to real-world units for accurate wound contraction assessment.

### 2.8. Scanning electron microscopy (SEM) for dual-species biofilm visualization on coverslips and wounds

To examine the formation of dual-species biofilms by *S. aureus* strain 6 and *A. baumannii* strain 1, scanning electron microscopy (SEM) was utilized on both coverslips and wound tissues [23]. This imaging was specifically conducted on untreated diabetic rats with infected wounds (control group) to verify the presence of established biofilms prior to initiating any treatment procedures.

Following biofilm development, both the coverslips and wound samples were carefully rinsed three times with sterile distilled water to remove non-adherent materials. The samples were then fixed in a solution of 2.5% glutaraldehyde prepared in 1 × phosphate-buffered saline (PBS) for three hours at room temperature. After fixation, the samples were rinsed three more times with distilled water and subjected to post-fixation using 1.5% osmium tetroxide for one hour. This was followed by another triple rinse with distilled water. The specimens were then gradually dehydrated using a series of ethanol solutions with increasing concentrations (20% to 100%), each step lasting 10 minutes. Once fully dehydrated, the samples were mounted on conductive copper SEM adhesive tape, coated with a thin layer of gold nanoparticles, and visualized using a scanning electron microscope (MIRA3, TESCAN Co., Czechia).

### 2.9. CFU counting method for bacterial quantification in wound samples

To quantify the bacterial load of *A. baumannii* and *S. aureus* in wound tissues, the colony-forming unit (CFU) method was employed. Sampling was conducted on days 1, 4, 8, 12, and 14 following wound induction. When signs of infection became apparent, sterile swabs were used to collect material from the wound surface. The collected material was weighed and then placed into a flask containing 1 mL of sterile normal saline per gram of tissue to prepare a bacterial suspension. Serial dilutions of the suspension were made, and 100 µL from each dilution was plated onto chocolate agar. The agar plates were incubated at 37°C for 24 hours, after which colony numbers were counted. To differentiate and quantify the individual bacterial loads, colonies grown on chocolate agar were subsequently transferred to Leeds Acinetobacter selective agar for *A. baumannii* and mannitol salt agar for *S. aureus*. This procedure allowed accurate determination of CFU per gram of tissue (CFU/g) for each species. These details were added to clarify how the two species were distinguished and quantified under different treatment conditions [24].

### 2.10. Real-time PCR analysis of biofilm-related gene expression

To assess the expression of biofilm-associated genes *icaA* in *S. aureus* and *bap* in *A. baumannii*, wound tissue samples were collected from each of the five groups on day 7 post-excision. The collected tissue was homogenized using a mechanical tissue homogenizer under sterile conditions to ensure efficient RNA extraction. The homogenized tissue was processed for total RNA extraction using the RNX-plus Mini Kit (Sinaclon Co, Iran), following the manufacturer’s protocol. To remove any residual genomic DNA, the extracted RNA was treated with DNase I prior to cDNA synthesis. RNA concentration, purity, and integrity were assessed, and 1 µg of RNA was used to synthesize complementary DNA (cDNA) with a cDNA Synthesis Kit (Parstous Biotechnology Co, Iran), according to the manufacturer’s instructions. Gene expression was quantified using the 2X Q-PCR Master Mix, with 2 µL of cDNA and 1 µL of each specific primer for *icaA*, *bap*, and 16S rRNA, in a total reaction volume of 20 µL. The amplification was performed on a real-time PCR system (LightCycler^®^ 96 Instrument, Roche, United States). The primers for *icaA*, *bap*, and 16S rRNA were derived from previously published studies [25,26]. Negative controls, including no-template controls (NTC) and no-reverse-transcriptase (No-RT) controls, were included in each run to ensure the absence of contamination and genomic DNA amplification. The relative gene expression levels were determined using the Ct method, with *16S rRNA* serving as the internal control for each bacterial species.

### 2.11. Histological evaluation

For histological assessment, tissue samples were collected from the wound sites on days 1, 4, 7, and 14 post-wounding to assess the effects of treatments on wound healing progression. The specimens were immediately fixed in 10% formalin, then processed routinely and embedded in paraffin. Sections of 5 µm thickness were prepared using a microtome and subsequently stained with hematoxylin and eosin (H&E) to evaluate the microscopic structure [27].

The histological analysis focused on several key parameters’ indicative of wound healing progression. Scab and crust formation were assessed as markers of the initial inflammatory process and served as a protective barrier during the early stages of healing. Granulation tissue and fibroplasia formation was examined based on collagen deposition and neovascularization, which are essential for tissue repair and regeneration... Re-epithelialization was assessed by determining the restoration and continuity of the epithelial layer, which indicates effective wound closure and functional recovery [28].

### 2.12. Statistical analysis

The data are presented as the mean ± standard deviation from three independent replicates. Statistical analysis was conducted using GraphPad Prism version 10.4.1. One-way ANOVA was applied to compare the microbial load between different treatment groups, with pairwise comparisons made using the Tukey test. A p-value of < 0.05 was considered statistically significant.

## 3. Result

### 3.1. In-vitro dual-species biofilm formation

The in-vitro formation of dual-species biofilms by *S. aureus* and *A. baumannii* was successfully established on microtiter plate wells. OD analysis confirmed the development of robust biofilms by both bacterial strains [29].

### 3.2. Dual-species biofilm formation on coverslips and its analysis

**Fig 1:**
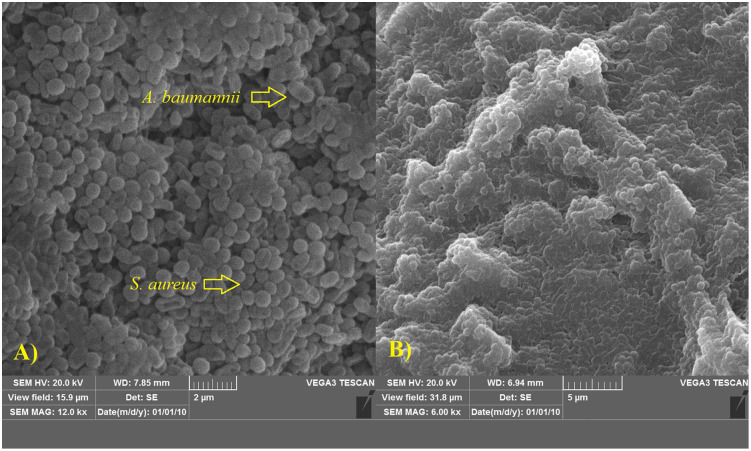
Visualization of dual-species biofilm formation by *Acinetobacter baumannii* and *Staphylococcus aureus* using Scanning Electron Microscopy (SEM). **(A)** Biofilm formation on coverslips, demonstrating bacterial adhesion and structural integrity. **(B)** Biofilm development on a wound model in diabetic rats, highlighting microbial colonization in a physiologically relevant environment.

The presence of a confluent stain on the coverslip surface confirmed biofilm formation. Additionally, SEM further verified the development of a dual-species biofilm. The SEM images revealed the presence of coccobacillus-shaped bacteria, including *A. baumannii*, alongside cocci-shaped bacteria, such as *S. aureus* (Fig 1A).

### 3.3. Establishment of Type II diabetes in rats

**Fig 2:**
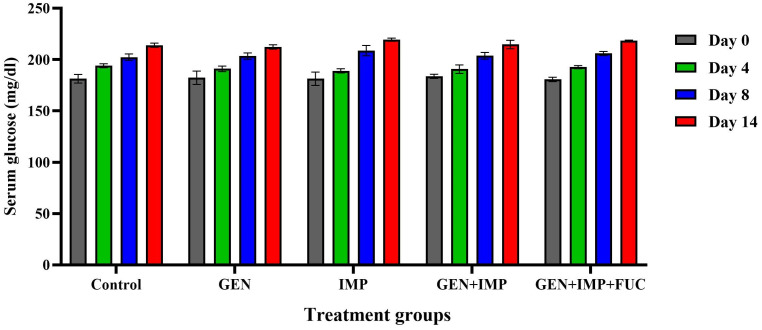
Blood glucose levels measured at different time points throughout the experimental period in rats. GEN: gentamicin; IMP: imipenem; FUC: fucoidan.

Streptozocin administration successfully induced diabetes in all rats. The dosage was carefully adjusted to maintain fasting blood glucose levels between 150 mg/dL and 200 mg/dL, preventing mortality from severe hyperglycemia. A slight increase in blood glucose levels was observed throughout the experimental period (Fig 2).

### 3.4. Excisional wound model in diabetic rats

A mature biofilm was observed on the wound surface within 48 hours following the application of the coverslip and bacterial broth culture. The infected tissue exhibited a grayish discoloration, along with visible pus and exudate production. Microbiological analysis at the 48-hour mark indicated bacterial loads ranging from 5.41 to 6.21 log CFU per gram of wound tissue. Additionally, SEM imaging confirmed the presence of a dual-species biofilm, showing coccobacillus-shaped bacteria, including *A. baumannii*, alongside cocci-shaped bacteria, such as *S. aureus* (Fig 1B).

**Fig 3:**
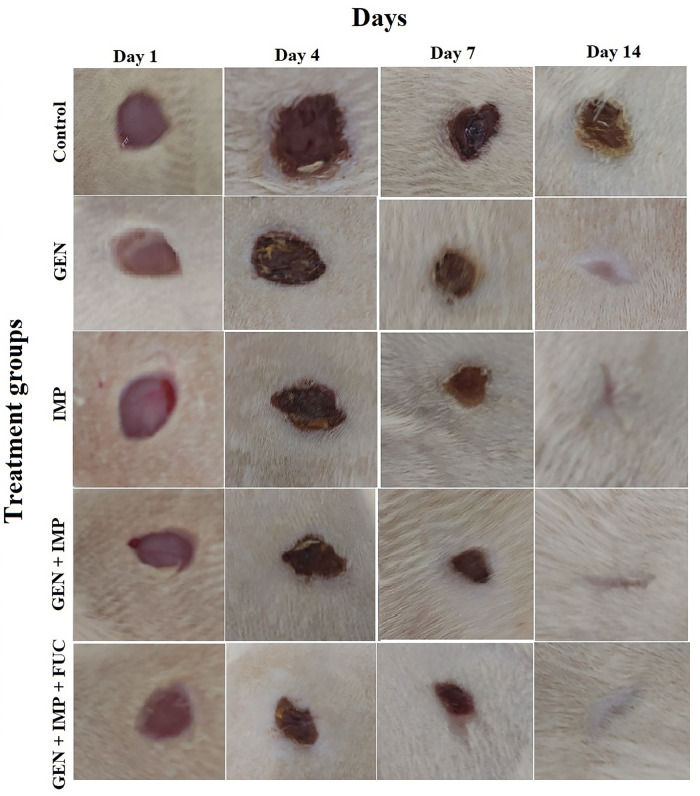
Wound healing in diabetic rat models infected with dual-species biofilm. This figure illustrates the wound diameter and wound closure process over a period of 14 days in diabetic rat models infected with a dual-species Staphylococcus aureus and Acinetobacter baumannii biofilm. The experimental groups included diabetic rats with wound infection and no treatment (control group), diabetic rats treated with gentamicin (GEN) alone, diabetic rats treated with imipenem (IMP) alone, diabetic rats treated with a combination of gentamicin and imipenem, and diabetic rats treated with a combination of gentamicin, imipenem, and fucoidan (FUC). The data demonstrate the progressive changes in wound size under each treatment regimen, highlighting the comparative effectiveness of single-drug and combination therapies in promoting wound healing in infected diabetic wounds.

As shown in Fig 3, the wound size in the control group (diabetic rats with wound infection and no treatment) did not close after 14 days, indicating persistent infection and impaired healing. In contrast, complete wound closure was observed in all treated groups by day 14. Notably, treatment with the combination of gentamicin, imipenem, and fucoidan resulted in the best response, with the most rapid wound closure and significant reduction in wound diameter. This was followed by treatment with a combination of gentamicin and imipenem. This combination also showed a markedly enhanced healing effect compared to treatment with gentamicin or imipenem alone. Moreover, the speed of wound healing and shrinkage was greater with the combination therapies (combination of gentamicin/ imipenem, and combination of gentamicin/ imipenem/ fucoidan) than with single-drug treatments, highlighting the synergistic benefits of combination therapy in accelerating wound repair in diabetic rats infected with dual-species *S. aureus* and *A. baumannii* biofilm.

### 3.5. Bacterial quantification in wound samples (CFU Counting)

**Fig 4:**
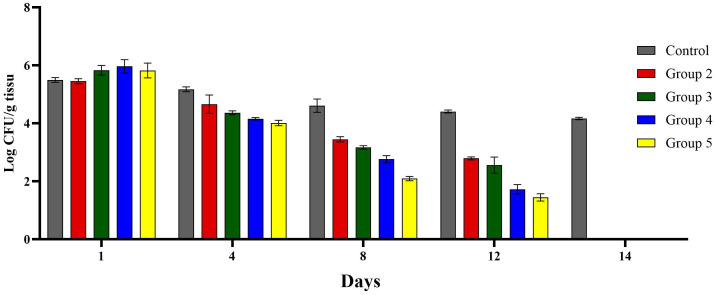
Healing of excision wounds in a dual-species biofilm infection model. The figure shows the bacterial load in wounded tissue across experimental groups: Group 1 – diabetic control (wound infection without treatment); Group 2 – treated with gentamicin; Group 3 – treated with imipenem; Group 4 – treated with gentamicin and imipenem; Group 5 – treated with gentamicin, imipenem, and fucoidan.

On day 12, the bacterial load (CFU/g tissue) was significantly reduced in diabetic rats treated with the combination of gentamicin, imipenem, and fucoidan. This substantial reduction compared to the control group highlighted the effectiveness of the triple therapy in combating the infection. Treatment with a combination of gentamicin and imipenem resulted in the second-highest reduction in bacterial load, followed by treatment with gentamicin or imipenem alone. The progressive decline in bacterial counts across these treatments highlights the superiority of combination therapies over single-drug treatments (Fig 4).

### 3.6. Real-time PCR analysis of biofilm-related gene expression

**Fig 5:**
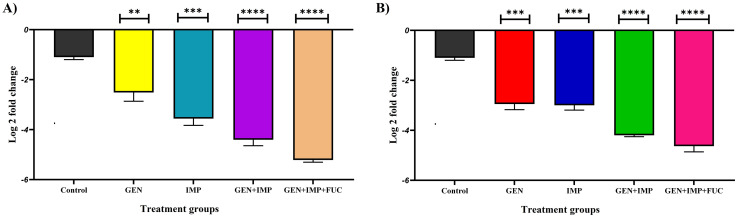
Expression levels of biofilm-associated genes. (A) *icaA* gene in *S. aureus* and **(B)** *Bap gene* in *A. baumannii* following treatment with gentamicin, imipenem, and fucoidan in the wound tissues of diabetic rats across different groups. Data are presented as mean ± SD (n = 3), with statistical significance indicated as ***P < 0.001 and ****P < 0.0001.

Real-time PCR analysis of wound tissue samples collected on day 7 post-excision revealed a significant reduction in the expression of biofilm-associated genes, specifically *icaA* in *S. aureus* and *bap* in *A. baumannii*, across all treatment groups (p < 0.005). The most pronounced decrease in gene expression was observed in diabetic rats treated with the combination of gentamicin, imipenem, and fucoidan. This was followed by treatment with gentamicin + imipenem, then imipenem alone, and the least reduction was observed in diabetic rats treated with gentamicin alone (Fig 5).

### 3.7. Histological evaluation

**Fig 6:**
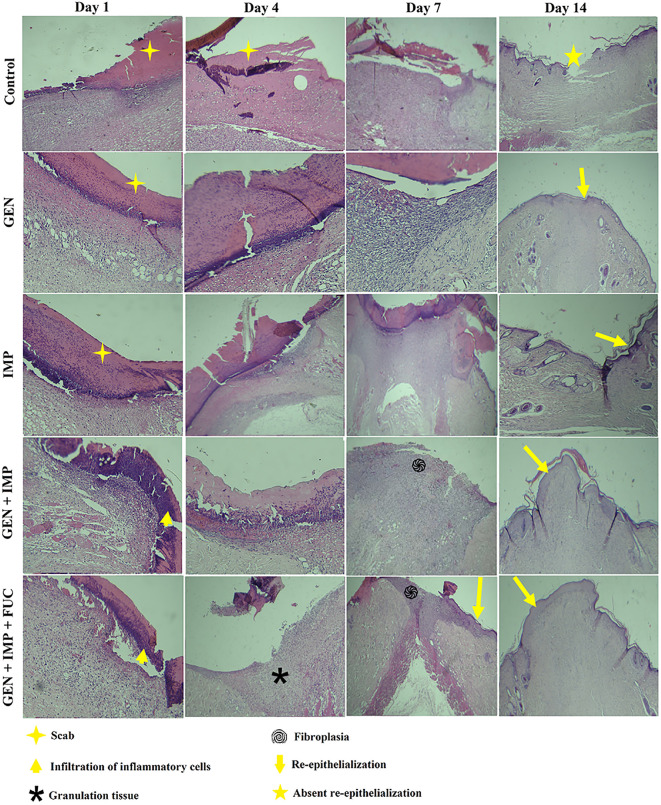
Histological evaluation of wound healing in diabetic rat wound models treated with different therapies. This figure presents the histological analysis of wound tissues collected from diabetic rats infected with dual-species Staphylococcus aureus and Acinetobacter baumannii biofilm after treatment. The examined groups included untreated diabetic rats (control group), rats treated with gentamicin (GEN) alone, rats treated with imipenem (IMP) alone, rats treated with a combination of gentamicin and imipenem, and rats treated with a combination of gentamicin, imipenem, and fucoidan (FUC). Tissue sections were evaluated for, granulation tissue, fibroplasia formation, and re-epithelialization.

Histological assessments of wound sections are presented in Fig 6. On day 1, scab formation was evident in all experimental groups. By day 4, the control group still retained residual scab, indicating delayed healing, while the Gentamicin and Imipenem monotherapy groups demonstrated persistent crust with minimal granulation tissue development. In contrast, wounds in the triple therapy group exhibited clear scab detachment with evident granulation tissue formation, marking progression towards tissue repair. On day 7, scab detachment was observed in all groups. The Gentamicin and Imipenem combination group showed marked fibroplasia and neovascularization with initiation of re-epithelialization from wound margins, whereas the triple therapy group displayed substantial wound contraction and re-epithelialization covering nearly one-third of the wound area, indicating a superior healing trajectory. By day 14, all groups showed considerable healing compared to baseline; however, small open wound areas were still present in the control group. Complete epithelialization was noted in each treatment group, with wound contraction in the Gentamicin and Imipenem combination group exceeding that of the monotherapy groups, while the triple therapy group exhibited the most advanced healing outcome, characterized by complete re-epithelialization

## 4. Discussion

Diabetic foot ulcers (DFUs) remain among the most challenging complications of diabetes mellitus, primarily due to delayed healing, chronic infection, and a high risk of amputation [15].

The hyperglycemic environment in diabetic patients compromises immune responses, reduces angiogenesis, and promotes microbial colonization, thereby facilitating chronic wound infection [30]. Among the pathogens commonly isolated from these wounds, *S. aureus* and *A. baumannii* play significant roles in infection persistence and treatment failure [31,32]. Both organisms are well-recognized for their ability to form robust biofilms, which not only protect them from host immune defenses but also render them highly resistant to antimicrobial agents [33,34]. Biofilm-associated infections are particularly problematic in diabetic wounds, as the biofilm matrix limits antibiotic diffusion and sustains prolonged inflammation [35]. The emergence of multidrug-resistant (MDR) strains has further complicated treatment strategies, necessitating alternative or adjunctive therapeutic approaches to effectively manage such infections.

Our previous in vitro investigations demonstrated the inhibitory and eradication potential of gentamicin, imipenem, and fucoidan, both individually and in combination, against dual-species biofilms formed by *S. aureus* and *A. baumannii* [16]. Therefore, translating these findings into an in vivo diabetic wound model is essential to better understand their therapeutic relevance under complex physiological conditions. Building upon those findings, the present in vivo study aimed to evaluate the therapeutic efficacy of these agents in a diabetic rat wound model infected with a dual-species biofilm, offering translational insights relevant to clinical application.

In this study, the excisional wound model in diabetic rats successfully established persistent infections, as evidenced by wound tissue exudation, delayed healing, and high bacterial loads. Notably, untreated control wounds remained open after 14 days, confirming the chronic and refractory nature of such dual-species biofilm infections in diabetic wounds. In contrast, all treated groups showed wound closure by day 14, with the combination therapy of gentamicin, imipenem, and fucoidan demonstrating the most rapid and complete wound healing. This finding is consistent with our previous in vitro results, where combination therapy exhibited enhanced/additive effects against biofilm formation and viability [16,29].

The bacterial load analysis further underscored the superior efficacy of combination therapy. Diabetic rats treated with gentamicin, imipenem, and fucoidan exhibited a substantial reduction in bacterial counts compared to all other groups. This reduction is clinically relevant, as high bacterial burden is strongly associated with delayed healing and persistent inflammation in chronic wounds [36]. These findings align with studies such as that of Lee et al., which demonstrated synergistic effects of fucoidan with gentamicin and ampicillin against oral pathogens, and Choi et al., who reported enhanced oxacillin and ampicillin activity against MRSA when combined with fucoidan [37,38]. Such synergy is likely attributable to fucoidan’s multifaceted bioactivity, including disruption of biofilm matrix integrity, interference with bacterial adhesion, and enhancement of antibiotic penetration.

Real-time PCR analysis further revealed a marked downregulation of biofilm-associated genes, specifically *icaA* in *S. aureus* and *bap* in *A. baumannii*, particularly in the triple therapy group. This suggests that the combination of gentamicin, imipenem, and fucoidan not only exerts bactericidal effects but also inhibits biofilm gene expression pathways critical for structural maintenance and pathogenicity. Similar findings were reported by Mani et al., who showed suppression of biofilm-related genes alongside antimicrobial activity in treated pathogens [39].

Histological evaluation provided additional evidence of the therapeutic benefits of combination therapy. On day 14, the triple therapy group displayed complete re-epithelialization, mature granulation tissue formation, and advanced wound contraction compared to monotherapy or dual therapy groups. Such histopathological improvements indicate enhanced progression through normal wound-healing phases. This indicates an accelerated transition through the phases of wound healing. Previous studies have shown that imipenem and gentamicin act synergistically by targeting bacterial cell wall synthesis and protein synthesis, respectively, while fucoidan contributes to wound healing via potential anti-inflammatory, antioxidant, and pro-angiogenic effects, further promoting fibroplasia and epithelialization [14,40,41].

The observed enhanced healing with combination therapy can be mechanistically explained by several factors. Firstly, the broad-spectrum bactericidal activities of gentamicin and imipenem effectively reduce bacterial burden, while fucoidan may disrupt biofilm matrix integrity, enhancing antibiotic penetration [42]. Secondly, fucoidan potentially modulates the local wound microenvironment by reducing inflammation and oxidative stress, both of which are critical barriers to healing in diabetic wounds [43]. Lastly, fucoidan’s structural similarity to glycosaminoglycans allows it to potentially influence cellular processes such as migration, proliferation, and angiogenesis, thereby accelerating tissue repair [44]. Consistent with previous studies, these combined actions likely contribute to the enhanced therapeutic outcomes observed in our in vivo model.

## 5. Conclusion

This study’s findings emphasize the enhanced therapeutic potential of combining gentamicin, imipenem, and fucoidan to treat diabetic wounds co-infected with *S. aureus* and *A. baumannii* biofilms. While the observed effects demonstrate additive or enhanced benefits rather than proven synergy, the combination therapy effectively accelerates wound healing, reduces bacterial load, and downregulates biofilm-associated genes. The inclusion of fucoidan provides additional potential supportive mechanisms as an adjunct to conventional antibiotics. These results provide a strong foundation for further investigation of combination therapies for diabetic foot ulcers and highlight the need for studies to elucidate the precise mechanisms underlying fucoidan’s contribution.
